# Lymphocytosis Increases the Immunity to Cancer

**Published:** 1915-11

**Authors:** 


**Lymphocytosis Increases the Immunity to Cancer,** is the conclusion of John B. Murphy and John J. Morton of the Rockefeller Institute, presented to the Washington Academy of Science. They suggest that injection with extract of lymphoid tissues might increase the immunity. Here is, at least, a scientific possibility for the control of cancer, irrespective of its paraistic or endogenic nature. Ordinary clinical examinations can easily determine whether cancer occurs in persons having relative lymphocytosis.

